# Supplementary material for: Modeling glioblastoma heterogeneity as a dynamic network of cell states
Source: Mol Syst Biol. 2021 Sep 16;17(9):e10105. doi: 10.15252/msb.202010105 (PMC8444284; doi:10.15252/msb.202010105)
Supplement: Supplementary file 5 — Source Data for Figure 3 [file MSB-17-e10105-s001.zip › Figure3A_sourcedata/GSEA_3065/hallmarks_state2.GseaPreranked.1623416269049/HALLMARK_PEROXISOME.html]

Details for gene set HALLMARK\_PEROXISOME[GSEA]

|  || Dataset | state2 |
| Phenotype | NoPhenotypeAvailable |
| Upregulated in class | na\_pos |
| GeneSet | HALLMARK\_PEROXISOME |
| Enrichment Score (ES) | 0.39452168 |
| Normalized Enrichment Score (NES) | 1.2280374 |
| Nominal p-value | 0.14345992 |
| FDR q-value | 0.19491675 |
| FWER p-Value | 0.997 |
Table: GSEA Results Summary

  

Fig 1: Enrichment plot: HALLMARK\_PEROXISOME      
 Profile of the Running ES Score & Positions of GeneSet Members on the Rank Ordered List

  

| PROBE | GENE SYMBOL | GENE\_TITLE | RANK IN GENE LIST | RANK METRIC SCORE | RUNNING ES | CORE ENRICHMENT || 1 | TOP2A |  |  | 15 | 0.596 | 0.1163 | Yes |
| 2 | IDH2 |  |  | 218 | 0.272 | 0.1486 | Yes |
| 3 | YWHAH |  |  | 221 | 0.271 | 0.2020 | Yes |
| 4 | MSH2 |  |  | 266 | 0.244 | 0.2456 | Yes |
| 5 | CNBP |  |  | 599 | 0.164 | 0.2426 | Yes |
| 6 | ITGB1BP1 |  |  | 750 | 0.144 | 0.2550 | Yes |
| 7 | IDI1 |  |  | 878 | 0.130 | 0.2672 | Yes |
| 8 | SOD1 |  |  | 1028 | 0.117 | 0.2745 | Yes |
| 9 | ERCC1 |  |  | 1065 | 0.114 | 0.2932 | Yes |
| 10 | CTPS1 |  |  | 1223 | 0.103 | 0.2968 | Yes |
| 11 | FDPS |  |  | 1297 | 0.098 | 0.3084 | Yes |
| 12 | ECI2 |  |  | 1422 | 0.091 | 0.3132 | Yes |
| 13 | ISOC1 |  |  | 1470 | 0.088 | 0.3256 | Yes |
| 14 | SMARCC1 |  |  | 1516 | 0.086 | 0.3378 | Yes |
| 15 | PABPC1 |  |  | 1589 | 0.082 | 0.3463 | Yes |
| 16 | PRDX5 |  |  | 1642 | 0.080 | 0.3566 | Yes |
| 17 | HRAS |  |  | 1748 | 0.075 | 0.3602 | Yes |
| 18 | PRDX1 |  |  | 1763 | 0.074 | 0.3733 | Yes |
| 19 | FIS1 |  |  | 1818 | 0.072 | 0.3818 | Yes |
| 20 | CTBP1 |  |  | 1897 | 0.068 | 0.3870 | Yes |
| 21 | ALDH9A1 |  |  | 1951 | 0.066 | 0.3945 | Yes |
| 22 | PEX2 |  |  | 2116 | 0.061 | 0.3890 | No |
| 23 | SIAH1 |  |  | 2552 | 0.046 | 0.3517 | No |
| 24 | HSD17B4 |  |  | 2829 | 0.038 | 0.3298 | No |
| 25 | CRAT |  |  | 2856 | 0.038 | 0.3345 | No |
| 26 | SOD2 |  |  | 3366 | 0.026 | 0.2854 | No |
| 27 | SLC25A4 |  |  | 3452 | 0.025 | 0.2813 | No |
| 28 | VPS4B |  |  | 3659 | 0.021 | 0.2635 | No |
| 29 | SLC25A17 |  |  | 3827 | 0.018 | 0.2493 | No |
| 30 | ERCC3 |  |  | 3877 | 0.018 | 0.2476 | No |
| 31 | FADS1 |  |  | 3941 | 0.016 | 0.2441 | No |
| 32 | ECH1 |  |  | 4011 | 0.015 | 0.2398 | No |
| 33 | PEX5 |  |  | 4355 | 0.010 | 0.2051 | No |
| 34 | BCL10 |  |  | 4493 | 0.007 | 0.1919 | No |
| 35 | CLN6 |  |  | 4714 | 0.004 | 0.1692 | No |
| 36 | HMGCL |  |  | 4735 | 0.004 | 0.1678 | No |
| 37 | CDK7 |  |  | 4953 | 0.000 | 0.1447 | No |
| 38 | IDE |  |  | 4954 | 0.000 | 0.1448 | No |
| 39 | ABCD3 |  |  | 5091 | -0.001 | 0.1306 | No |
| 40 | TSPO |  |  | 5245 | -0.004 | 0.1150 | No |
| 41 | HSD17B11 |  |  | 5658 | -0.011 | 0.0733 | No |
| 42 | SCP2 |  |  | 5857 | -0.014 | 0.0549 | No |
| 43 | ACAA1 |  |  | 6129 | -0.019 | 0.0297 | No |
| 44 | GNPAT |  |  | 6186 | -0.020 | 0.0277 | No |
| 45 | PEX6 |  |  | 6188 | -0.020 | 0.0314 | No |
| 46 | IDH1 |  |  | 6208 | -0.020 | 0.0334 | No |
| 47 | PEX11B |  |  | 6273 | -0.021 | 0.0307 | No |
| 48 | MLYCD |  |  | 6396 | -0.023 | 0.0224 | No |
| 49 | ACOT8 |  |  | 6528 | -0.026 | 0.0135 | No |
| 50 | PEX13 |  |  | 7009 | -0.037 | -0.0305 | No |
| 51 | ELOVL5 |  |  | 7020 | -0.037 | -0.0242 | No |
| 52 | CAT |  |  | 7094 | -0.039 | -0.0243 | No |
| 53 | DHCR24 |  |  | 7171 | -0.041 | -0.0242 | No |
| 54 | ACSL1 |  |  | 7456 | -0.049 | -0.0448 | No |
| 55 | SLC35B2 |  |  | 7486 | -0.050 | -0.0380 | No |
| 56 | ABCC5 |  |  | 7542 | -0.052 | -0.0337 | No |
| 57 | SLC23A2 |  |  | 7559 | -0.052 | -0.0251 | No |
| 58 | CLN8 |  |  | 7561 | -0.052 | -0.0149 | No |
| 59 | ACSL4 |  |  | 7960 | -0.065 | -0.0444 | No |
| 60 | LONP2 |  |  | 8123 | -0.073 | -0.0474 | No |
| 61 | MVP |  |  | 8452 | -0.093 | -0.0640 | No |
| 62 | GSTK1 |  |  | 8455 | -0.093 | -0.0458 | No |
| 63 | ACOX1 |  |  | 8525 | -0.098 | -0.0337 | No |
| 64 | CADM1 |  |  | 8914 | -0.137 | -0.0480 | No |
| 65 | ATXN1 |  |  | 8996 | -0.148 | -0.0273 | No |
| 66 | RDH11 |  |  | 9108 | -0.167 | -0.0060 | No |
| 67 | SEMA3C |  |  | 9242 | -0.209 | 0.0211 | No |
Table: GSEA details [plain text format]

  

Fig 2: HALLMARK\_PEROXISOME: Random ES distribution      
 Gene set null distribution of ES for **HALLMARK\_PEROXISOME**
